# Mitigation of methane gas emission in rice by drip irrigation

**DOI:** 10.12688/f1000research.20945.1

**Published:** 2019-11-28

**Authors:** Theivasigamani Parthasarathi, Koothan Vanitha, Sendass Mohandass, Eli Vered

**Affiliations:** 1 VIT School of Agricultural Innovations and Advanced Learning (VAIAL), Vellore Institute of Technology, Vellore, Tamil Nadu, 632014, India; 2 Department of Crop Physiology, Tamil Nadu Agricultural University, Coimbatore, Tamil Nadu, 641003, India; 3 Netafim Irrigation Ltd, Kibbutz Mahal, Israel

**Keywords:** Aerobic rice, Drip irrigation, Methane, CO2 eq-emission, Water productivity

## Abstract

**Background:** Rice farming faces major challenges, including water limitation, drought and climate change in the current scenario of agriculture. Among the innovative water-saving techniques, drip irrigation is a forerunner, with maximized water-saving potential, increased grain yield and methane mitigation.

**Methods:** A field experiment was conducted comprising four different drip irrigation practices: (i) sub-surface drip irrigation (SDI) with 1.0 litre per hour (lph) discharge rate emitters (DRE) (SDI+1.0 lph DRE) (ii) SDI+0.6 lph DRE, (iii) surface drip irrigation (DI) with 1.0 lph discharge rate emitters (DI+1.0 lph DRE), (iv) DI+0.6 lph DRE and were compared with (v) a conventional flood aerobic irrigation (considered conventional).

**Results:** The estimated grain yield of rice was found to be 23.5%, 20.3%, and 15.1% higher under SDI+1.0 lph DRE, SDI+0.6 lph DRE and DI+1.0 lph DRE practices, respectively, than the conventional method. A water saving of 23.3% was also observed for all drip practices compared with conventional practices. Seasonal methane emission flux declined 78.0% in the drip methods over the conventional irrigation: better mitigation than previously reported values (alternate wetting and drying (47.5%) and system of rice intensification (29.0%) practices). Continuous soil aeration and enhanced soil methanotrophs (P<0.05) limit the peak methane emission in rice during the flowering phase in drip irrigation, which is reflected in the methane emission flux values. Consequently, the equivalent CO _2_ (CO _2_-eq) emissions and yield-scaled CO _2_ eq-emission were found to be significantly lower in SDI (43.8% and 49.5%, respectively), and DI (25.1% and 26.7%, respectively) methods as compared with the conventional that ensures better methane mitigation and future climate-smart rice production systems.

**Conclusions: **Drip irrigation could reduce the cumulative methane emission in aerobically grown rice. SDI + 1.0 lph DRE practice can be applied in areas with inadequate water availability and effective in reducing the CO _2_-eq emission with better yield than conventional.

## Introduction

Agriculture was estimated to account for 10–20% of manmade greenhouse gas (GHG) emissions (5.1–6.1 Gt CO _2_-eq year ^-1^ by 2030) worldwide ( Smith *et al.*, 2008; Tubiello *et al.*, 2013). Rice fields are a major source of agricultural methane (CH _4_) emissions ( Malyan *et al.*, 2016), contributing 20–40 Tg CH _4_ year ^-1^ with a global emission of 52% ( Sun *et al.*, 2016). In India, rice cultivation covers about 44 million ha, the largest rice-producing area in Asia. To ensure food security for the exponential population, expanding the cropping area will increase methane emission. Therefore, reducing methane gas emission from the rice eco-system is the foremost preventive measure for a check-in global warming. Altering water level maintenance, an important parameter in measuring methane emission rate, by shifting water level from 15 cm to 10 cm could reduce 26.8% of total methane emissions in the rice field and supports a green eco-system ( Sandin, 2005). So, effective water management practices, like midseason drainage, intermittent irrigation, system of rice intensification, alternate wetting and drying, direct dry seeding and aerobic rice cultivation, have the possible potential to mitigate methane emission for irrigated rice cultivation ( Bronson *et al.*, 1997; Feng *et al.*, 2013; Trost *et al.*, 2013). The system of rice intensification (SRI) practice reduces total methane emissions by 29.0% ( Rajkishore *et al.*, 2013), the value for alternate wetting and drying method was 44.0% ( Bouman *et al.*, 2005; Bouman *et al.*, 2007; Oo *et al.*, 2018a; Oo *et al.*, 2018b; Setyanto *et al.*, 2018) and for aerobic rice practice was 51.0% ( Joshi *et al.*, 2009; Jain *et al.*, 2014; Keppler *et al.*, 2006; Sharma *et al.*, 2016) compared with flooded rice cultivation. Although above-water management strategies show reduced methane emissions, consumption of more water for initial field setup and surface mode flood irrigation during an entire rice-growing cycle reduces the water productivity ( Geethalakshmi *et al.*, 2011) of the rice crop. By 2025, Asia’s 130 million ha of irrigated rice area may experience physical and economic water scarcity ( Tuong & Bouman, 2003). In addition, India would need to produce up to 156 million tonnes of rice by 2030 ( Dass *et al.*, 2016) to feed its 1,523 million population. So, it is necessary to develop alternative irrigation strategies to mitigate methane emission as well as to improve the rice yield with limited water ( Bouman *et al.*, 2005; Reis *et al.*, 2018; Yang & Zhang, 2010).

The drip irrigation system for rice is a water-saving concept that allows the rice farmers to utilize water effectively through root-zone irrigation ( Parthasarathi *et al.*, 2018), which may lead to more rice crop seasons in a year. Also, drip irrigation has scope to mitigate methane emissions in the rice ecosystem. Drip irrigation to rice altered root traits ( Parthasarathi *et al.*, 2017), improved the water productivity ( He *et al.*, 2013; Parthasarathi *et al.*, 2013; Parthasarathi *et al.*, 2018) and nutrient use efficiency ( Rajwade *et al.*, 2018), and reduced pollution of the environment ( Adekoya *et al.*, 2014). Drip irrigation kept the field in the condition similar to aerobic/upland throughout the growing season ( Adekoya *et al.*, 2014). Rice ecosystems managed by drip irrigation have been scarcely reported with regards to methane emissions and mitigation potential. The effect of drip irrigation on soil environment, growth, yield and water productivity of rice remains unexplained.

We, therefore, hypothesized that the drip irrigation practice would allow for improved water saving, increased yield and the potential to mitigate methane release in the rice ecosystem. We tested this hypothesis and examined the drip irrigation practices (i.e. sub-surface drip irrigation, surface drip irrigation, 1.0 and 0.6 lph discharge rate emitters) by conducting the field experiment in aerobic rice. We measured the methane emission, soil environment, rice growth, yield, water productivity, and microbial abundance. The results demonstrated that drip irrigation can be adapted to maintain more oxidative aerobic soil condition in rice crop.

## Methods

### Study background

**Figure 1.**
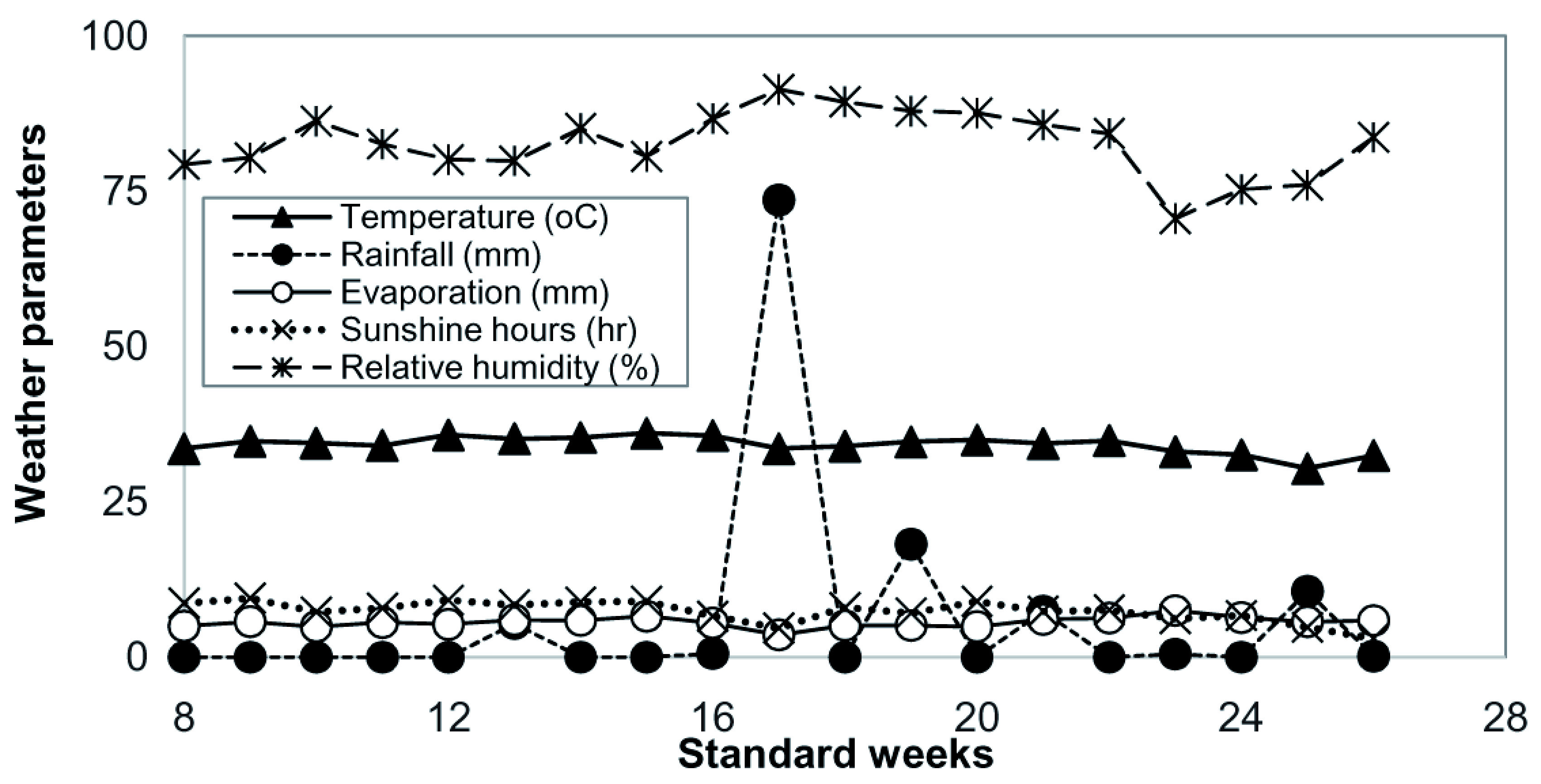
Weather condition [Air temperature (°C), Rainfall (mm), Evaporation (mm), Sunshine hours (hr) and relative humidity (%)] prevailing during the cropping period.

The drip irrigation with methane emission experiment was conducted during summer season 2014 in the wetlands of Tamil Nadu Agricultural University, Coimbatore, Tamil Nadu, India (located at 110 N latitude, 770 E longitude and at an altitude of 426.8 m above mean sea level). The prevailing agro-ecological conditions during the cultivation period were an average temperature of 34.2/23.3°C (max/min), sunshine hours of 7.3 hrs day ^-1^ and total evaporation of 750.4 mm with total precipitation of 118.6 mm ( Figure 1). Soil samples were collected in the field and soil physio-chemical properties were analysed and given in Table 1. Drip experiment was carried out using ADT (R) 45 rice variety (parentage: IR 50/ADT 37) that grows widely in the Cauvery delta zone of Tamil Nadu, India.

**Table 1.** Soil physical and chemical properties of the experimental site.

pH	EC (dS m ^-1^)	Organic carbon (%)	Available N (kg ha ^-1^)	Available P (kg ha ^-1^)	Available K (kg ha ^-1^)
8.1	0.74	0.75	326	24.6	358

### Drip system

The drip system was installed in the field with the help of Netafim Irrigation, Israel. The drip irrigation was supplied through 40 mm OD PVC pipes by 7.5 HP motors from bore well and pressure maintained in the system was 1.2 kg cm ^-2^. The drip treatments tested were sub-surface drip irrigation (SDI) with 1.0 litre per hour (lph) discharge rate emitters: i) SDI+1.0 lph DRE (SDI with 0.6 lph discharge rate emitters); ii) SDI+0.6 lph DRE (surface drip irrigation (DI) with 1.0 lph discharge rate emitters); iii) DI+1.0 lph DRE (DI with 0.6 lph discharge rate emitters) and iv) DI+0.6 lph DRE.

These were arranged in a randomized block design with three replications per method. Drip irrigation lateral pipes were laid out at a distance of 0.8 m, emitters placed at 0.3 m distance for DI and SDI. Besides, the laterals placed at a depth of 15 cm below the soil surface for the SDI. Rice plants under drip irrigation system were irrigated at 125% pan evaporation (PE) using the Open Pan Evaporation (PE) values from a USWB Open Pan Evaporimeter. Scheduling of irrigation for the drip methods was conducted by working out effective rainfall using water balance method ( Dastane, 1974). A conventional flood aerobic irrigation practice was considered the conventional method; these plots were kept unsaturated and the surface irrigated at 30-mm depth when irrigation water/cumulative pan evaporation (IW/CPE) ratio reached 1.25. A graphical description of irrigation practices, crop evapotranspiration (ET) and soil moisture prevailed during the experiment is given in the *Extended data*, Supplementary Figures 1 and 2 ( Parthasarathi, 2019). Regarding crop management, recommended cultivation practices for aerobic rice were followed ( TNAU Crop production guide, 2014). Application of pre-emergence herbicide, pendimethalin 30% EC at 1.25 kg a.i. ha ^-1^ at 3 days after sowing (DAS) and two-time hand weeding at 30 and 45 DAS have controlled the weeds. A recommended fertilizer dose of 150:50:50 kg ha ^-1^ N:P:K was supplied as fertigation in the form of water-soluble fertilizers. Fertigation was applied through a venturi flume at weekly intervals. For the conventional irrigation method, the entire dose of P was applied basally before sowing. In the case of N, the recommended dose was given at basal, tillering, booting and 50% flowering; K was given in two equal splits at basal and panicle initiation stages.

### Methane sampling and analysis

**Figure 2.**
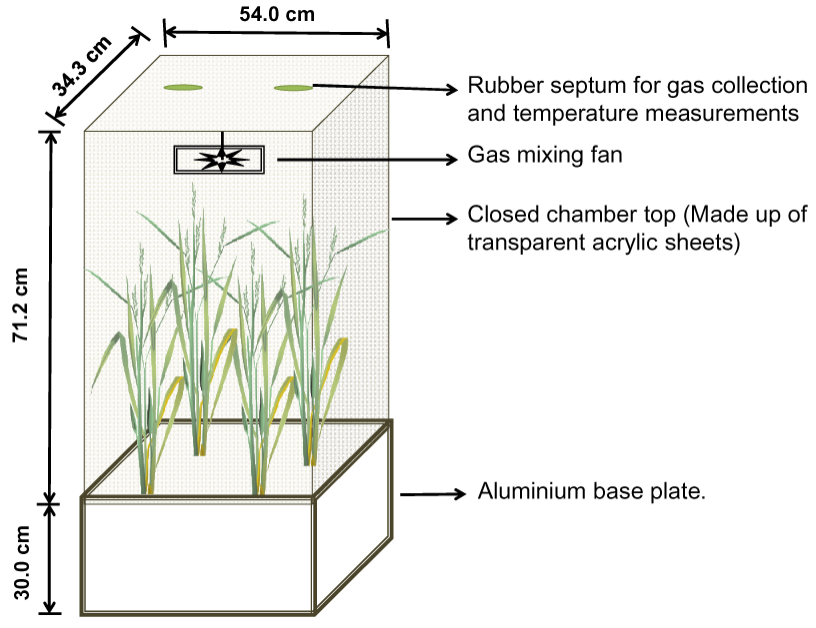
Diagram of closed diffusion chambers system used to collect methane emission in the rice field.

The sampling of methane gas was performed using the closed chamber technique ( Minamikawa *et al.*, 2015). The chamber with rice plants is illustrated in Figure 2. The chamber was placed in between the laterals and 15 cm far from the emitters. Inside the chamber, an electric fan was installed to circulate the air. Samples were collected from 10:00 to 12:00 at 10, 30, 50, 70, 90 and 110 DAS. The gas samples were withdrawn from the top of the chamber using 50-ml gas-tight syringes at 0, 10, 20 and 30 min after putting the chamber in its place. Air inside the chamber was thoroughly mixed by flushing the syringe five times before collection of the gas sample. The sample gases were transferred to 15 ml vacuum glass vials with a rubber stopper and kept cool and dark until analysis.

The temporal increment of methane concentration inside the chamber was measured in terms of methane flux ( Hutchinson & Mosier, 1981). Collected gas samples were analysed using gas chromatography with flame ionization detector (FID). The following formula used to calculate methane gas emitted and concentration of gas denoted as mg m ^-2^ hr ^-1^:

Total methane emissions (mg m ^-2^ hr ^-1^) = [(Ps × Cs / Pstd) × Vv / Va] × Vhx A × H.

Where Ps = peak area for sample in gas chromatography; Cs = standard methane gas concentration (mg L ^-1^ ); Vv = vial volume (ml); Vh = headspace volume of the chamber, i.e., [Chamber length*breadth*height] (ml); Va = air volume sampled (ml); A = chamber area covered (m ^2^); H : enclosure period (hr); Pstd : standard peak area in gas chromatograph. The equivalent CO _2_ (CO _2_-eq) emission for total methane was calculated using the following modified equation ( Oo *et al.*, 2018a): CO _2_-eq = [TCH _4_ × 34], where CO _2_-eq is the total amount of equivalent CO _2_ emission (kg CO _2_-eq ha ^-1^), TCH _4_ is the total amount of methane emission (kg ha ^-1^), 34 is the global warming potentials for methane to CO _2_ over a 100-year time horizon ( IPCC, 2013).

### Soil characteristics

The soil physical measurements were recorded during methane gas collection. Soil redox potential (Eh) and pH were measured by portable Eh meter (EHS-120; Fujiwara Scientific Company Co., Ltd, Tokyo, Japan) with platinum-tipped electrodes and pH meter (Lutron model pH 212; Sunshine Instruments, India), respectively. Dissolved oxygen content (mg L ^-1^) of rice rhizosphere was measured with the analytical apparatus SJG-203A (Shanghai Leici Manufacturers, Shanghai, China). Enumeration of methanotrophic methane-oxidizing bacteria (MOB) was isolated and quantified during flowering phase based on the method of Graham *et al.* (1992). The soil samples were collected, diluted as suspension and analysed to 10 ^-2^ to 10 ^-7^ levels by 1-mL suspension in tubes under ambient air condition.

### Plant characteristics

The plant height (cm) and total dry matter accumulation (g m ^-2^) were measured during methane gas collection. The plant volume and root oxidizing power were taken from tagged plants during the flowering stage of rice. The total plant volume was calculated as the sum of root and shoot volume. Root and shoot volume was recorded by using the water displacement method ( Bridgit & Potty, 2002). The values were expressed in cm ^3^ hill ^-1^. The oxidizing power of roots was determined by the method of Ota (1970) and the activity expressed as μg g ^-1^ hr ^-1^. At the end of the experiment, replication wise harvesting was done for each treatment at the net plot (2.4 × 7.0 m). The yield of rice was measured at 14% moisture level and yield expressed in kg ha ^-1^. Harvest index (HI) was calculated by using the formula of Yoshida *et al.* (1971) and expressed in percentage.

### Water use

Water use was calculated by the sum of irrigation water applied (mm) and the effective rainfall (mm) during the cropping period ( Dastane, 1974). Water productivity was calculated as the grain weight produced per unit of water input ( Yang *et al.*, 2005) and expressed as g grains kg ^-1^ of water.

### Statistical analysis

Randomized block design (RBD) analysis was carried out on various parameters (Water productivity, methane flux rate, cumulative methane, CO _2_-eq emissions, Yield-scaled CO _2_-eq emission, Grain yield, Straw yield, Harvest index, plant height, Total dry mass accumulation (TDMA), soil pH, soil Eh, Dissolved oxygen, plant volume, Root oxidizing power, Methanotrophs population) as per the procedure suggested by Gomez & Gomez (1984). The coefficient of determination (R ^2^) was made between methanotrophs population and methane flux. Whenever the treatment differences were found significant, critical differences were worked out at 5% probability level. Analysis of variance (ANOVA) was carried out for the recorded mean data using JMP, 2007 (SAS Institute, Cary, NC) software; appropriate standard errors of the means (SEM) and Fisher’s Least Significant Difference at a significance of P ≤ 0.05 was calculated.

## Results and discussion

The SDI and DI systems were installed in the field with 1.0 lph and 0.6 lph DRE to the aerobic rice plants. The drip response results on methane gas emission, CO _2_-eq emission, soil pH, soil redox potential, soil methanotrophs population from the rice ecosystem, plant height, total dry mass, yield, water productivity of rice were compared with the conventional flood aerobic irrigation practice; this section discusses the notable results. Data concerning methane emissions and weather for each group is available as *Underlying data* ( Parthasarathi, 2019).

### Effect of drip irrigation on seasonal methane flux

**Figure 3.**
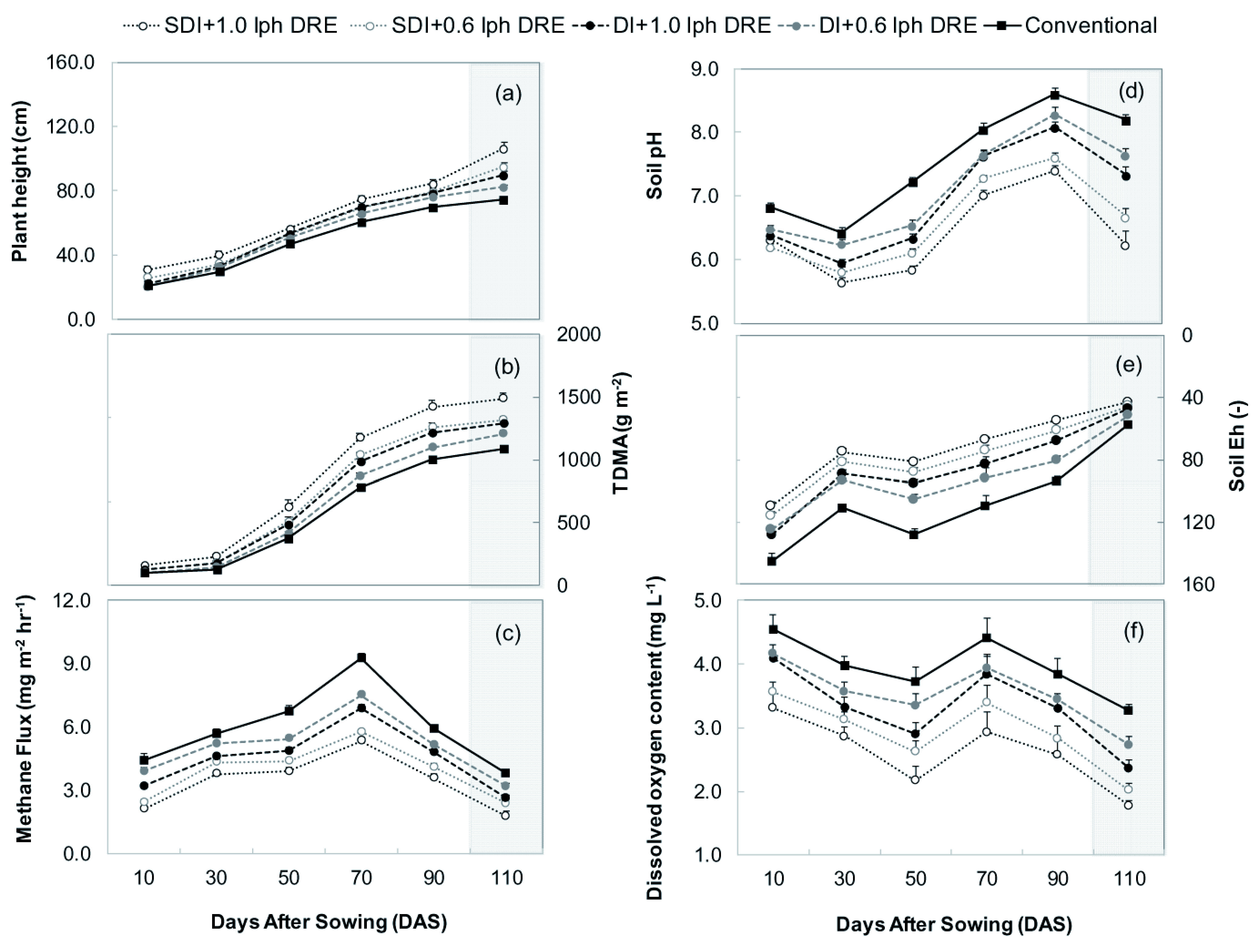
Effect of drip treatments on rice growth, change in methane flux, soil pH, soil Eh (redox potential) and dissolved oxygen content of drip-irrigated soil environment. Error bars indicate standard error of the mean (n = 6). Shading indicates the period after post-flowering phase (100 DAS). SDI, sub-surface drip irrigation; DI, surface drip irrigation; DRE, discharge rate emitters; Conventional, conventional flood aerobic irrigation.

Seasonal methane flux pattern was observed to be similar in conventional and drip treatments ( Figure 3c), and the flux increased gradually at the early rice-growing stage but declined at the end of the growth period ( Figure 3c). A recent report by Oo *et al.* (2018a) on the alternate wetting and drying (AWD) water-saving method in rice emitted methane gas with two peak emissions during the vegetative phase. Contrarily, drip and conventional aerobic irrigation practices had a single emission peak during the flowering phase (70 DAS), particularly at 10 days before and after anthesis. This is because the aerobic condition lacked the dominant pathway of direct methane emission by lesser aerenchyma pore spaces in the root (see *Extended data*, Supplementary Information 2 ( Parthasarathi, 2019)). This is the reason for the reduced methane transport through rice shoots from soil to the atmosphere and was well explained by Bhattacharyya *et al.* (2019) in rice. Contrary to the sharp decline in methane flux at harvest phase in flooded paddy ( Minamikawa *et al.*, 2015), drip and conventional aerobic irrigated rice showed a gradual and slow decline influx after 100 DAS (shaded area in Figure 3). The decrease in methane transport capacity of rice plants under an aerobic environment is the possible explanation for methane reduction after flowering ( Figure 3c) and the response was reverse for flooded paddies ( Minamikawa *et al.*, 2015). These different responses to irrigation conditions between flooded and aerobic environment explain the significance of the drip system on methane mitigation potential ( Table 2).

**Table 2.** Water usage, water productivity and methane flux (mg m ^-2^ h ^-1^) by different drip irrigation treatments and conventional aerobic rice growing seasons. Numbers in the table represent means ± standard deviation (n =3). Letters indicate significant differences (P<0.05) among means according to an ANOVA.

Treatments	Total water applied (mm)	Irrigation water (mm)	Effective rainfall (mm)	Water productivity (g grains kg ^-1^ of water)	Methane flux rate (mg m ^-2^ h ^-1^)
SDI+1.0 lph DRE	591.1	509.7	81.5	0.82±0.03 ^A^	3.37±0.2 ^D^
SDI+0.6 lph DRE	591.1	509.7	81.5	0.77±0.04 ^AB^	3.87±0.2 ^CD^
DI+1.0 lph DRE	591.1	509.7	81.5	0.72±0.02 ^B^	4.50±0.2 ^BC^
DI+0.6 lph DRE	591.1	509.7	81.5	0.70±0.02 ^B^	4.98±0.2 ^B^
Conventional	771.5	690.0	81.5	0.54±0.02 ^C^	6.00±0.3 ^A^

SDI, sub-surface drip irrigation; DI, surface drip irrigation; DRE, discharge rate emitters; Conventional, conventional flood aerobic irrigation.

The SDI at 1.0 and 0.6 lph DRE practices found a significant reduction in methane emission during vegetative to post-flowering phases due to discontinuous soil aeration near the root zone and lack of carbon substrate for methane production (root exudates) during the growing season. Similar reduction reported in the midseason drained paddy field ( Jiao *et al.*, 2006) and these were in agreement with Yan *et al.* (2005) and Sander *et al.* (2015), who reported an average methane reduction of 40–60% by multiple soil aerations.

**Figure 4.**
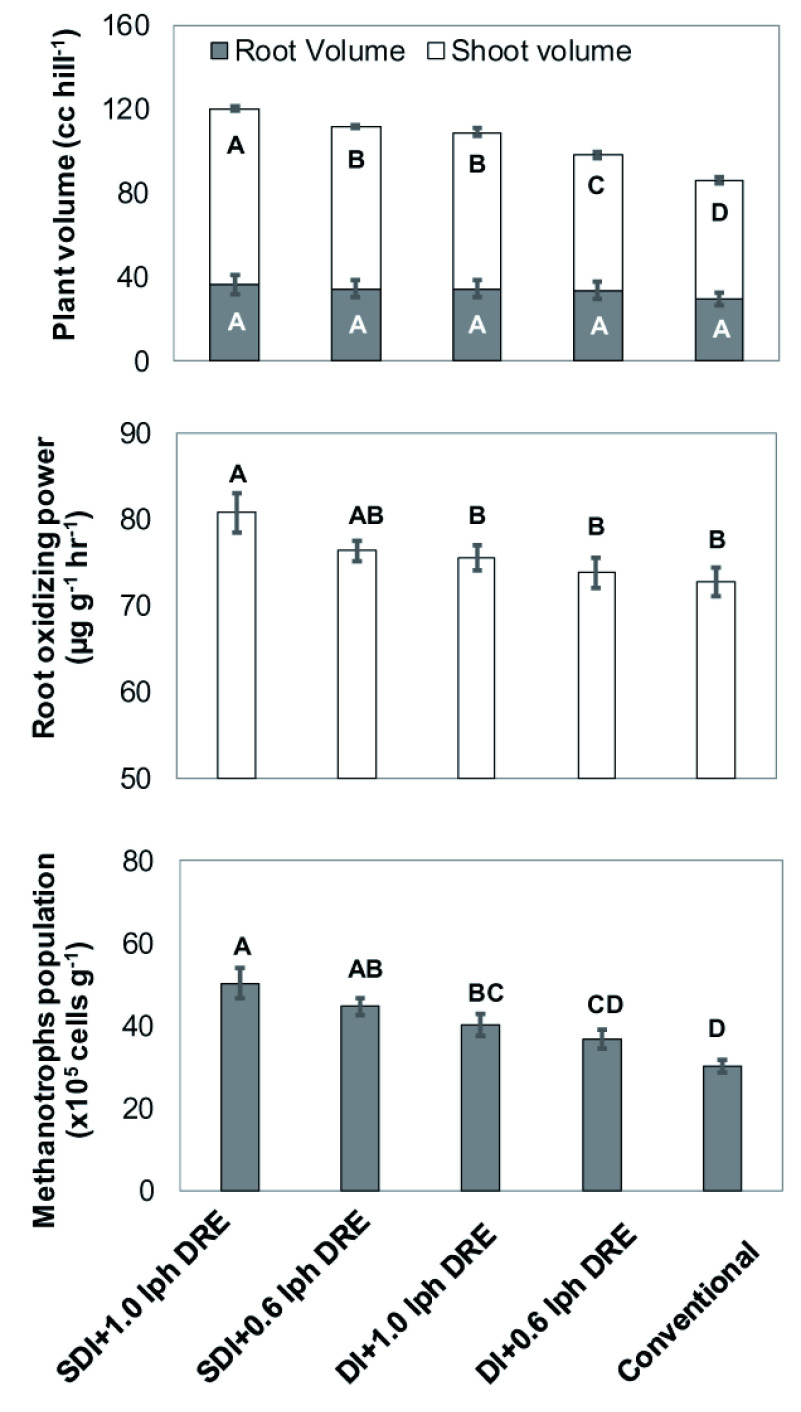
Effect of drip irrigation treatments on change in plant volume (root+shoot), root oxidizing power and soil methanotrophs population. Error bars indicate standard error of the mean (n = 6). Different letters denote significant differences among means derived using an ANOVA and student test. SDI, sub-surface drip irrigation; DI, surface drip irrigation; DRE, discharge rate emitters; Conventional, conventional flood aerobic irrigation.

**Figure 5.**
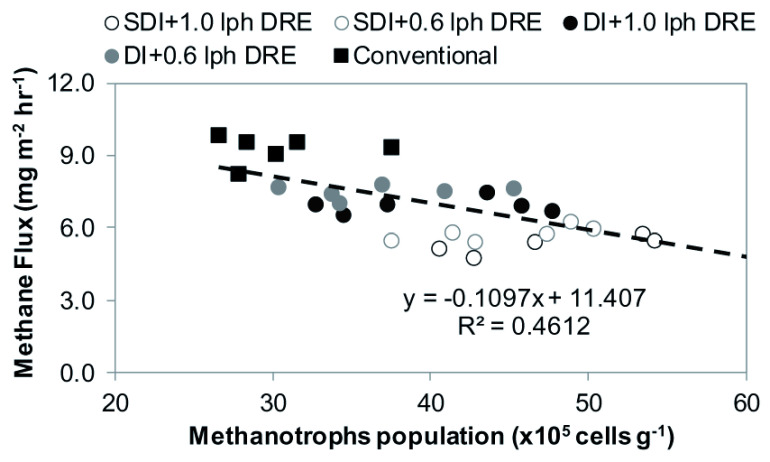
Methane emission as a function of methanotroph population under drip and conventional treatments during flowering. The black line shows a regression line (y=-0.1097x+11.407). The r ^2^ is the coefficient of determination. SDI, sub-surface drip irrigation; DI, surface drip irrigation; DRE, discharge rate emitters; Conventional, conventional flood aerobic irrigation.

 As hypothesized, a significant reduction (P < 0.05) in the rate and cumulative methane flux was observed under drip irrigation methods over the conventional. Lesser methane flux rate ( Table 2), cumulative methane ( Table 3) emitted in SDI + 1.0 lph DRE (3.37 mg m ^-2^ h ^-1^ and 97.2 kg ha ^-1^, respectively) treatment with greater mean significance over DI + 1.0 lph DRE (4.50 mg m ^-2^ h ^-1^ and 129.6 kg ha ^-1^, respectively) and conventional (6.00 mg m ^-2^ h ^-1^ and 172.9 kg ha ^-1^, respectively). The drip treatments (SDI+1.0 lph DRE, SDI+0.6 lph DRE, DI+1.0 lph DRE, DI+0.6 lph DRE) might have directed to more oxygen penetration to the root zone that inhibits the methanogenic bacteria by methane oxidation. However, the dissolved oxygen content declined significantly in drip practice over the conventional methods; we discuss the reason for the lower values in the up-coming section. Soil methanotrophic bacteria oxidizing methane gas using molecular oxygen in drained paddy soil ( Jiao *et al.*, 2006) was the reason for the above reduction in methane flux rate, cumulative methane flux under drip environment. This response was further confirmed an increased in methanotroph population of 39.8% in SDI+1.0 lph DRE, 32.2% in SDI+0.6 lph DRE (44.7 ×10 ^5^ cells g ^-1^) followed by 24.8% in DI+1.0 lph DRE over the aerobic zones of the rhizosphere ( Figure 4). The regression line ( Figure 5) represents a negative slope (y=-0.1097x+11.407, R ^2^ = 46.1) or the relationship between methanotrophs population and methane emission that indicates the drip practice reduces the methane by increased methanotrophs. Similarly, drip practices inhabited the aerobic interfaces of the methanogenic environment, that inhibit methanogenesis by killing the methanogenic bacteria ( Hanson & Hanson, 1996) and these processes were influenced by the soil physio-chemical properties, and plant growth parameters ( Luo *et al.*, 2013).

**Table 3.** Effect of drip system on cumulative methane, CO _2_-eq emission, yield-scaled CO _2_-eq emission, and rice yield. Numbers in the table represent means ± standard deviation (n =3). Letters indicate significant differences (P<0.05) among means according to an ANOVA.

Treatments	Cumulative methane (kg ha ^-1^)	CO _2_-eq emissions (kg CO _2_ ha ^-1^)	Yield-scaled CO _2_-eq emission (kg CO _2_-eq t ^-1^)	Yield Observation		
				GY	SY	HI (%)
				(Kg ha ^-1^)	(Kg ha ^-1^)	
SDI+1.0 lph DRE	97.2±6.0 ^D^	3304.8±202.5 ^D^	300.1±18.4 ^C^	4489±111.9 ^A^	7297±243.3 ^A^	38.1±0.31 ^A^
SDI+0.6 lph DRE	111.4±5.9 ^CD^	3788.9±200.7 ^CD^	348.1±18.4 ^C^	4305±69.0 ^A^	7234±250.5 ^AB^	37.4±0.60 ^AB^
DI+1.0 lph DRE	129.6±6.8 ^BC^	4406.4±231.6 ^BC^	435.6±22.8 ^B^	4038±125.6 ^B^	6943±304.3 ^AB^	36.8±0.70 ^AB^
DI+0.6 lph DRE	143.5±6.7 ^B^	4877.8±230.9 ^B^	499.5±23.8 ^B^	3783±60.0 ^B^	6719±295.7 ^AB^	36.1±0.75 ^BC^
Conventional	172.9±8.9 ^A^	5879.7±303.0 ^A^	594.2±30.6 ^A^	3430±65.2 ^C^	6562±110.3 ^B^	34.3±0.66 ^C^
**Mean**	**130.9**	**4451.5**	**435.5**	**4009.2**	**6950.9**	**36.6**

SDI, sub-surface drip irrigation; DI, surface drip irrigation; DRE, discharge rate emitters; Conventional, conventional flood aerobic irrigation.

### Effect of drip irrigation on rice growth and surrounding environment

The drip irrigation practices had a considerable change in rice growth in terms of plant height (29.4%) and total dry mass accumulation (TDMA) (26.9%) than conventional systems. These were significantly increased under SDI and DI methods, irrespective of emitter discharge rates, over the conventional method. These results were consistent with the findings of previous studies, which found that the drip irrigation had improved the production potential of rice under non-flooded irrigation ( He *et al.*, 2013 and Parthasarathi *et al.*, 2015). The total dry mass of rice did not differ significantly between drip irrigation and conventional irrigation up to 30 DAS, although after anthesis (70 DAS), it significantly improved under SDI + 1.0 lph DRE practice ( Figure 3a). This was mainly related to enhanced dry mass accumulation after anthesis, which explained by Parthasarathi *et al.* (2015) and Parthasarathi *et al.* (2018), who reported drip irrigation increased the plant height and leaf photosynthetic rates of rice during post-anthesis. TDMA showed a moderate change during the early growth and vegetative phase of rice under SDI and DI methods ( Figure 3b), which reasoned for the lesser emission of methane over conventional. These results corroborated with the findings of Arafa *et al.* (2009) in wheat under SDI system.

Higher dry mass and lower root volume of rice facilitated oxygen transport into the rhizosphere, stimulating methane oxidation ( Ma *et al.*, 2010). Similarly, Figure 4 illustrates unaffected root zone volume and increased shoot volume under drip irrigation favours methane oxidation in comparison with the conventional method. This might be due to the favourable balance between root and shoot translocation of assimilates, nutrients and water ( Kludze *et al.*, 1993). Higher shoot volume increased the contact surface between roots that enhanced the methane oxidation rates under drip irrigated rice environment and were in agreement with methane oxidation studies in rice by Zhao *et al.* (2013). SDI + 1.0 lph DRE treatment significantly improved the root oxidase activity (80.7 µg g ^-1^ hr ^-1^) than the conventional (72.7 µg g ^-1^ hr ^-1^) ( Figure 4). This indicated an overall higher oxidation status and might have accelerated methane oxidation in the root rhizosphere, which eventually reduced the methane gas emission ( Kong *et al.*, 2009).

Among the irrigation treatments, drip-irrigated rice had higher (P<0.05) soil Eh than conventional. Superior Eh levels reasoned for the lesser emission (P<0.05) of methane under SDI+1.0 lph DRE drip practice ( Figure 3e). Present results followed the study of Oo *et al.* (2018a), who reported water management practice improved the soil redox potential in flooded rice. Dorau & Mansfeldt (2015) and Minamikawa *et al.* (2015) reported that higher Eh was resulted by the low pH in soil solution and the similar was observed in SDI+1.0 lph DRE followed by SDI+0.6 lph DRE and DI+1.0 lph DRE practices ( Figure 3d). This acidic shift might also be due to the supply of water-soluble fertilizers by drip fertigation. This low soil pH could be the reason for the altered balance between CH _4_ and CO _2_ under drip irrigation by soil organic matter decomposition ( Kongchum, 2005). Thus, low soil pH had a considerable effect, but this was alongside soil oxygen properties, which are likely to be responsible for reduced methane emissions ( Kajiura *et al.*, 2018). SDI + 1.0 lph DRE treatment registered lesser (P<0.05) dissolved oxygen (DO) in soil than the conventional during all the phenophases ( Figure 3f). This condition led to an increase in the soil oxygen concentration that leads to inhibition of soil organic matter oxidation which directly and indirectly, inhibited the methanogenesis ( Kato *et al.*, 2012; Zhou *et al.*, 2014). Soil organic carbon (SOC) of oxygen-enhanced soil was oxidized into carbon dioxide (CO _2_) instead of methane (CH _4_), which ultimately reduced the root zone methane formation ( Sun *et al.*, 2016). This contrasted with flooded rice ( Sharkawi *et al.*, 2009), where increased DO reduces the methane emission but DO in the rhizosphere was controlled by irrigation water quantity.

### Effect of drip irrigation on water usage, crop productivity, and CO _2_-eq emission

Under drip irrigation, the total water saving compared with conventional flood aerobic irrigation was 23.3% ( Table 2). The maximum saving of irrigation water with aerobic irrigation was 50% ( Peng *et al.*, 2006), with alternate wetting and drying was 47% ( Oo *et al.*, 2018a) and with the system of rice intensification was only 27.4% ( Suryavanshi *et al.*, 2013), relative to flooded rice. Higher water savings under drip irrigation over the conventional method was due to the supply of irrigation water being based on the evapotranspiration demand for rice. The water productivity of 0.82 g grains kg ^-1^ of water obtained under drip irrigation (SDI + 1.0 lph DRE) due to higher yield potential and substantially higher water productivity than the previous reports ( Bouman *et al.*, 2006; Kato *et al.*, 2009).

 Drip irrigation improves the grain yield of rice by 9.3-23.6% over the conventional flood aerobic irrigation. Higher grain yield (4489 kg ha ^-1^), straw yield (7297 kg ha ^-1^) and harvest index (38.1%) were obtained under SDI + 1.0 lph DRE practice, and it was significantly lower using the conventional method (3430 kg ha ^-1^, 6562 kg ha ^-1^ and 34.3%, respectively) ( Table 3). The yield of rice was significantly superior under SDI followed by DI over the conventional aerobic rice. Present yield response of rice in drip irrigation was due to better water and nutrients discharge (1.0 lph DRE followed by 0.6 lph DRE) to the root zone. These were in line with the recent work on drip-irrigated rice ( Parthasarathi *et al.*, 2018) along with plastic mulching ( He *et al.*, 2013). Higher harvest index of rice was due to the better balance between the source and sink under drip irrigation. This might be reasoned for the methane reduction ( Denier *et al.*, 2002) under drip environment.

The impact of methane emission calculated by the CO _2_-eq emission for the 100-year horizon was observed for each method. The SDI and DI treatments reduced the CO _2_-eq emission by 43.8% and 25.1% over conventional flood aerobic irrigation ( Table 3). This reduction found a better result than the recent report ( Oo *et al.*, 2018a) on the alternate wetting and drying method (19%-39% better than the conventional flooded method). The decrease in methane emissions by SDI and DI was due to effective depression in CO _2_-eq methane emission. Yield-scaled CO _2_-eq emission provides a measure of agronomic efficiency to mitigate climate change and future food production concerns ( Grassini & Cassman, 2012). The yield-scaled CO _2_-eq emissions were higher (594.2 kg CO _2_-eq t ^-1^) using the conventional method than SDI + 1.0 lph DRE practices (300.1 kg CO _2_-eq t ^-1^) due to their respective levels of methane emission rate. Therefore, the SDI + 1.0 lph DRE practice is recommended for efficient reduction in CO _2_-eq methane emission along with increased grain yield and water productivity, rather than the conventional flood aerobic irrigation.

## Conclusion

The present study demonstrated that drip irrigation practice can mitigate methane emissions and improved the growth and yield of rice when compared to conventional aerobic methods. The drip combinations SDI + 1.0 lph DRE, SDI + 0.6 lph DRE, DI + 1.0 lph DRE and DI + 0.6 lph DRE could reduce the cumulative methane emission in aerobic rice by diminishing the flux rate. So far, it has been impossible to control the soil redox condition of conventional aerobic irrigated rice soil, but the drip irrigation practice (SDI or DI with 1.0 or 0.6 lph DRE) may offer the solution. The better performance of drip irrigation (i.e. SDI+ 1.0 lph DRE) under aerobic conditions was also evident from their higher TDMA, root oxidase activity, soil methanotrophs population along with higher methane mitigation in comparison to the conventional. The SDI + 1.0 lph DRE practice can be applied in areas with inadequate water availability (i.e. where there are water shortages) for flooded rice production and the same practice is effective in reducing the CO _2_-eq emission and better grain yield than the conventional flood aerobic irrigation. In the case of climate change, drip irrigation systems have promise to ensure food security, while preserving irrigation water and mitigating methane gas emissions in rice. Further studies are required to test the methane-mitigating oxygen-nanobubble water ( Minamikawa *et al.*, 2015) under rice crop drip-irrigation, and to evaluate its mitigation potential regarding methane and other greenhouse gases.

## Data availability

### Underlying data

Open Science Framework: Methane Emission. https://doi.org/10.17605/OSF.IO/ZDY6U ( Parthasarathi, 2019).

This project contains the following underlying data:

- Raw data methane emission (data concerning methane emission for each experimental group)
- Raw data weather (data concerning the weather for each experimental group)

### Extended data

Open Science Framework: Methane Emission. https://doi.org/10.17605/OSF.IO/ZDY6U ( Parthasarathi, 2019)

This project contains the following extended data:

- Supplementary Information (1) (containing Supplementary Figures 1 and 2)
- Supplementary Information (2) (containing supplementary Figure 3 and Supplementary Table 1)

Data are available under the terms of the Creative Commons Attribution 4.0 International license (CC-BY 4.0).
