# Supplementary material for: Mitigation of methane gas emission in rice by drip irrigation
Source: F1000Res. 2019 Nov 28;8:2023. [Version 1] doi: 10.12688/f1000research.20945.1 (PMC8276196; doi:10.12688/f1000research.20945.1)
Supplement: Peer review history

# Peer review: Reviewer response for version 1

**DOI:** 10.5256/f1000research.23049.r87960

**Published:** 2021-07-09

**Authors:** Gerard Arbat

**Affiliations:** 1 Department of Chemical and Agricultural Engineering and Technology, University of Girona, Girona, Catalonia, 17003, Spain

The paper deals with methane emission, water use, and water productivity of drip irrigated rice compared with conventional flooded aerobic irrigation. The study includes surface and subsurface drip irrigation with two different flowrates. The main results of the paper are that drip irrigation reduces methane emissions and increases water productivity. The best option for this irrigation system is subsurface drip irrigation at a flowrate of 1 lph.

 My recommendation would be a "Minor revision". This revision may include the following changes:

1. Include the soil texture (%sand, silt and clay) in table 1, as it can affect the results obtained
2. Define 'standard weeks' in Fig. 1.
3. Show in Fig. 2 the exact location of the chamber, as it is described in Methane sampling and analysis section: “…placed in between the laterals and 15 cm from the emitters.”
4. In the section soil characteristics, the measurements are not only related to physical characteristics, please them describe accordingly.
5. In the section plant characteristics, define what a hill is.
6. In Fig.3, the vertical titles of figs. B and E are in different location than the other figures. Please be consistent.

Is the work clearly and accurately presented and does it cite the current literature?

Yes

If applicable, is the statistical analysis and its interpretation appropriate?

Yes

Are all the source data underlying the results available to ensure full reproducibility?

Yes

Is the study design appropriate and is the work technically sound?

Yes

Are the conclusions drawn adequately supported by the results?

Yes

Are sufficient details of methods and analysis provided to allow replication by others?

Yes

Reviewer Expertise:

Irrigation Engineer

I confirm that I have read this submission and believe that I have an appropriate level of expertise to confirm that it is of an acceptable scientific standard.

# Peer review: Reviewer response for version 1

**DOI:** 10.5256/f1000research.23049.r57226

**Published:** 2020-01-03

**Authors:** Prashantkumar S. Hanjagi

**Affiliations:** 1 Division of Plant Physiology, ICAR-Indian Agricultural Research Institute, New Delhi, Delhi, India

Thanks for asking me to review this article. The authors of the study have generated very good data to communicate the key message on mitigation of methane emission in rice by different irrigation methods. My recommendation would be in and around  "Minor revision". 

 My minor comments to the authors are:

1. In the Introduction text, the previously reported value for reduction in methane emissions due to alternate wetting and drying is 44.0%. But, in abstract it is being mentioned as 47.5%. Right value may be mentioned here. 
2. In methods section, treatments can be mentioned in a tabular form for better understanding of readers instead of writing in running text.
3. For figure 2, Authors could have presented the real set-up of closed diffusion chambers used to collect methane emission in the present study instead of diagrammatic representation.
4. Rewrite this sentence "This response was further confirmed an increased in methanotroph population of ..." in results section (page no 7). 

Is the work clearly and accurately presented and does it cite the current literature?

Yes

If applicable, is the statistical analysis and its interpretation appropriate?

Yes

Are all the source data underlying the results available to ensure full reproducibility?

Yes

Is the study design appropriate and is the work technically sound?

Yes

Are the conclusions drawn adequately supported by the results?

Yes

Are sufficient details of methods and analysis provided to allow replication by others?

Yes

Reviewer Expertise:

My research program is multidisciplinary in nature, combining physiology, molecular biology, and proteomics. Currently my research focuses on understanding the cellular and molecular mechanisms mediating the response(s) of plants to abiotic stress (Salt, drought, heat, lodging, etc). I have expertise in automated high-throughput plant phenotyping in getting insights into genomic performance and plant-environment-interactions through non-destructive analysis of phenotypic traits of the major crops like maize, rice, wheat, and pulses. Within my group, during past years, we have attempted to characterize the role of candidate genes (NHX1 & AVP1) in improving tolerance to salt stress tolerance in rice.

I confirm that I have read this submission and believe that I have an appropriate level of expertise to confirm that it is of an acceptable scientific standard.
